# Computational Design of a TCR-like Human V_H_ Domain Antibody That Specifically Recognizes a WT1-Derived Peptide Presented by Class I Major Histocompatibility Complex

**DOI:** 10.34133/csbj.0236

**Published:** 2026-10-15

**Authors:** Tae-Sung Oh, Seong-Woo Kim, SangPhil Ahn, Seonghyuk Suh, JooYeon Lee, YeongNam Bae, Hyejin Lee, Yong-Sung Kim, Bo-Seong Jeong, Byung-Ha Oh

**Affiliations:** ^1^ Therazyne, Inc., Daejeon, Republic of Korea.; ^2^ Department of Biological Sciences, Korea Advanced Institute of Science and Technology, Daejeon, Republic of Korea.; ^3^ Department of Molecular Science and Technology, College of Advanced Bioconvergence Engineering, Ajou University, Suwon, Republic of Korea.; ^4^ Graduate School of Engineering Biology, KAIST Institute for BioInnovation, Korea Advanced Institute of Science and Technology, Daejeon, Republic of Korea.

## Abstract

T cell receptor (TCR)-like antibodies that recognize a peptide–major histocompatibility complex (pMHC) can enable precise targeting of cancer cells, but developing single-domain binders with high specificity and affinity is challenging. Here, we report the computational design and experimental validation of a TCR-like human heavy chain variable (V_H_) domain antibody that specifically recognizes the Wilms’ tumor 1 protein (WT1)-derived peptide RMFPNAPYL presented by HLA-A*02:01/β2-microglobulin (RMF/HLA-A*02). Starting from the crystal structure of a Fab antibody bound to RMF/HLA-A*02, we repurposed the soluble trastuzumab-derived V_H_ scaffold to bind RMF/HLA-A*02. The resulting V_H_ domain, αRMF_V_H_, spans all 9 peptide residues and showed no binding to a control pMHC with a different peptide. Fusion of 1 or 2 αRMF_V_H_ molecules to human serum albumin domain III yielded monovalent and bivalent constructs with good biophysical properties. The monovalent construct bound selectively to RMF/HLA-A*02 with 81 nM affinity, and when expressed as part of a chimeric antigen receptor on T cells, it functioned as an antigen-recognition domain to selectively activate T cells in the presence of RMF/HLA-A*02-positive cells. The bivalent construct exhibited a dramatically increased apparent binding affinity of 0.37 nM. When both constructs were engineered into the bispecific T cell engager (TCE) format containing an anti-CD3 Fv, the bivalent TCE induced antigen-dependent cytotoxicity much more effectively than the monovalent TCE. These results demonstrate a viable strategy to develop high-specificity, peptide-focused TCR-like V_H_ domain antibodies that can be readily extended into potent bivalent TCEs for pMHC-targeted therapeutics.

## Introduction

Class I major histocompatibility complex (MHC) molecules are composed of a polymorphic human leukocyte antigen (HLA) heavy chain and a β2-microglobulin (β2M) light chain. Expressed on nearly all nucleated cells, they play a critical role in immune surveillance by enabling the detection of infected or malignant cells. Peptide–MHC (pMHC) antigens on the cell surface are continuously monitored by T cell receptors (TCRs). When a nonself peptide or an abnormal self-peptide is presented by a pMHC, it can activate the responsive T cells, which then target and kill the presenting cell [1].

Developing TCRs specific to target pMHCs is an important focus in research and pharmaceuticals. Two pMHC-targeting modalities have received clinical approval: tebentafusp, a TCR-derived bispecific T cell engager (TCE) targeting HLA-A*02:01-positive melanoma cells presenting a gp100-derived peptide [2], and afamitresgene autoleucel, an adoptive cell therapy targeting HLA-A*02:01-positive synovial sarcoma cells presenting a MAGE-A4-derived peptide [3].

A TCR-like antibody is a monoclonal antibody that mimics the specificity of a TCR in recognizing pMHCs. These antibodies combine the antigen-binding capacity of an antibody with the ability to recognize a specific peptide epitope presented by MHC molecules. Owing to their stability, versatility, and ease of manufacturability, TCR-like antibodies present a viable alternative to TCR-based therapies. Several TCR-like antibodies targeting pMHCs have been developed, showing promising clinical potential [4–7]. These antibodies cannot be directly used therapeutically unless their target antigens are exceptionally expressed on cancer cells. Instead, they need to be converted into specialized formats like T cell bispecific antibody [8], bispecific TCE [7], chimeric antigen receptor (CAR) [9], or STAR (synthetic TCRs and antigen receptors) in which the TCRα and TCRβ constant regions are fused to the V_L_ and V_H_ domains of a TCR-like antibody, respectively [10]. All of these formats function by activating T cells upon specific engagement with pMHC antigens.

Developing TCR-like antibodies with negligible cross-reactivity is challenging. This is because the size of the real antigen, the peptide displayed on an HLA molecule, is fractional compared to that of MHC. While interactions with the HLA scaffold can provide allele-specific anchoring and structural context, the large antigen-binding surface of a Fab may also engage conserved HLA surfaces, potentially increasing cross-reactivity with nontarget pMHC complexes. Nanobodies, with their smaller epitope-binding surfaces, could be advantageous TCR-like antibodies in reducing cross-reactivity, and a few TCR-like nanobodies have been developed and tested for effectiveness in the adoptive cell therapy format [11,12].

Beyond traditional library screening with positive and negative sorting, a strategy for reengineering preselected TCR-like antibodies was reported to target different pMHC antigens, enabling the isolation of new TCR-like antibodies [13]. In addition, the design of non-TCR-like, synthetic scaffolds to target specific pMHCs has also been reported [14,15].

Wilms’ tumor 1 protein (WT1) is a zinc finger transcription factor highly expressed in various cancers but rarely in normal adult cells [16–19]. WT1-derived peptides are presented by MHC as 9- or 10-mer peptides [20,21]. A 9-mer WT1 peptide (126-RMFPNAPYL-134, “RMF”) is presented by the HLA-A*02:01 subtype. TCRs or TCR-like antibodies have been developed that recognize RMF bound to HLA-A*02:01–β2M (for simplicity, denoted as RMF/HLA-A*02 hereafter) [8,22–24] and utilized for immunotherapeutic approaches against several cancers such as acute myeloid leukemia [8,25–27].

To date, no TCR-like nanobody specific for the RMF/HLA-A*02 complex has, to our knowledge, been reported. We report the repurposing of the anti-Her2 antibody trastuzumab’s V_H_ into a TCR-like single-domain antibody (sdAb), which selectively binds to RMF/HLA-A*02. Its potential for selective pMHC-targeted cancer therapy was evaluated in a chimeric antigen receptor T cell (CAR-T) and a TCE format.

## Materials and Methods

### Production of pMHC complexes

The RMF peptide (RMFPNAPYL) and the p53^(R175H)^ control peptide (HMTEVVRHC) were synthesized by GenScript. Peptide-loaded HLA-A*02:01 complexes were produced by protein refolding according to the reported procedures [28]. In brief, denatured HLA-A*02:01 and β2M were refolded in the presence of the synthetic peptide containing 20 mM Tris–HCl (pH 8.0), 400 mM l-arginine, 2 mM EDTA, 0.5 mM oxidized glutathione, 5 mM reduced glutathione, and 0.2 mM phenylmethylsulfonyl fluoride. The refolding reaction was incubated at 18 °C for 48 h, followed by concentration to 7.5 ml and buffer exchange using a PD-10 column equilibrated with a buffer solution containing 30 mM NaCl and 20 mM Tris–HCl (pH 8.0). The resulting solution was further purified by size-exclusion chromatography using a HiPrep 26/60 Sephacryl S-300 HR column (Cytiva), immobilized metal affinity chromatography (Ni-NTA), and anion-exchange chromatography using a Capto HiRes Q 5/50 column (Cytiva). The typical yield of final purified pMHC complexes was approximately 2 mg per 2-l refolding reaction.

### Production of Q2L-Fab and sdAb proteins

For the production of Q2L-Fab, DNA fragments encoding the V_H_–C_H1_ domain with a C-terminal 6×His tag and the V_L_–C_L_ domain of Q2L were synthesized (IDT) and cloned into a pTT5-derived expression vector (Addgene). The resulting plasmids were transfected into CHO-S cells (Gibco) at 6 × 10^6^ cells/ml using ExpiFectamine CHO (Gibco). Cells were cultured in ExpiCHO Expression Medium (Gibco) for 10 d. Supernatants were harvested by centrifugation at 12,000 × *g* for 1 h at 4 °C, diluted 1:1 with phosphate-buffered saline (PBS), and purified by Ni-NTA affinity chromatography (HisPur Ni-NTA resin, Thermo Scientific), followed by size-exclusion chromatography using a HiLoad 26/60 Superdex 75 column (Cytiva) equilibrated with PBS.

For the production of sdAbs, the gene encoding αRMF_V_H_ with a C-terminal 8×His tag was synthesized (IDT) and cloned into the pET-22b(+) vector (Novagen). The plasmid was transformed into *Escherichia coli* BL21 (DE3) RIPL cells (Novagen). Fusion constructs of αRMF_V_H_ and human serum albumin domain III (residues 384 to 585; HSA^DIII^) were similarly cloned. Protein expression was induced with 200 μM isopropyl β-D-1-thiogalactopyranoside (IPTG) at 18 °C overnight. Cells were harvested and resuspended in lysis buffer (150 mM NaCl, 20 mM Tris–HCl, pH 8.0), followed by sonication. The cleared lysate was purified using the same purification procedures as for Q2L-Fab.

### Crystallization, structure determination, and refinement

Optimized crystallization conditions for Q2L-Fab/RMF/HLA-A*02 were established by hanging-drop vapor diffusion at 20 °C, using 2-μl drops prepared by mixing equal volumes of protein and reservoir solutions. Q2L-Fab/RMF/HLA-A*02 crystals were obtained using the protein sample (8 mg/ml) and a reservoir solution composed of 8% (w/v) PEG 5000 MME, 5% (v/v) Tacsimate (pH 7.0), and 100 mM Hepes (pH 7.0). For cryoprotection, the crystals were transferred into the reservoir solution supplemented with 18.25% (v/v) glycerol and subsequently flash-frozen in liquid nitrogen. X-ray diffraction data were collected at beamline 5C of Pohang Accelerator Laboratory (Korea) and processed with HKL2000 [29]. Structures were determined by molecular replacement using an AlphaFold-Multimer-predicted model of Q2L-Fab and the crystal structure of RMF/HLA-A*02 (Protein Data Bank [PDB] entry: 3PHJ) [30] as probe molecules using PHENIX [31]. Electron densities for the 2 V_L_ domains were weak and vague unlike those for other parts of the complexes, which indicated that the position of V_L_ in the predicted Q2L-Fab structure was incorrect. The isolated V_L_ domain was then used for partial molecular replacement with fixed other parts of the complex using PHENIX. The correctly positioned models were refined using COOT [32] and PHENIX [31].

### Biolayer interferometry

Biolayer interferometry (BLI) experiments were performed using an Octet R8 system (Sartorius). Biotinylated RMF/HLA-A*02 at a concentration of 20 nM was loaded onto streptavidin biosensors (Sartorius). The loaded sensors were then equilibrated in Kinetics Buffer (Sartorius) for 60 s. To measure *K*_D_, αRMF_V_H_ and its HSA^DIII^-fusion constructs at 7 different concentrations were subjected to BLI runs with an association step (for 60 or 120 s) followed by a dissociation step (for 60 or 120 s). Biosensor tips were loaded with the biotinylated target protein (20 nM) to a loading response of ~0.5 nm. Sensorgrams for monovalent constructs were fitted using a 1:1 Langmuir binding model, whereas sensorgrams for the bivalent construct were analyzed using a 2:1 bivalent analyte model using the Octet BLI Analysis 12.2.2.4 software package (Sartorius). For specificity assessment, biotinylated p53^(R175H)^/HLA-A*02 and H2-Ab were used as controls, and the experimental procedure followed the same protocol as that used for affinity determination.

### Computational design

After superposing Tra V_H_ onto Q2L-Fab/RMF/HLA-A*02, the CDR3H loop of Tra V_H_ (residues Arg98 to Tyr109) was replaced with that of Q2L (residues Lys98 to Tyr105). Tra V_H_ residues corresponding to 2 CDRH2 paratope residues of Q2L were manually replaced (Y57E and R59L), and a bulky residue clashing with RMF/HLA-A*02 was mutated to alanine (Y33A). The resulting 4-chain model was first minimized in Cartesian coordinate space using the Rosetta MinMover protocol with the beta_nov16_cart score function to restore standard stereochemical geometry and was subsequently relaxed in torsional space using FastRelax (10 repeats) with the beta_nov16_cst score function (Rosetta version 2020.42.post.dev+13.master.3548d5e). No coordinate constraints were applied during these steps. The 10 resulting models were highly similar, with a standard deviation of 0.032 Å over all superposed Cα atoms. The mean Cα root-mean-square deviation values between the pre- and postrelaxation models were 0.40 Å for the framework, 0.36 Å for CDR1, 0.29 Å for CDR2, and 0.34 Å for CDR3. The output model with the best total score was selected. A single residue position (Ala33) was subsequently redesigned using Rosetta FastDesign (5 repeats) while applying coordinate constraints to the 3 pMHC chains with AddConstraintsToCurrentConformationMover. Among 20 design trajectories, threonine was the most frequently selected residue and was therefore chosen. Finally, Phe103, whose side chain was largely solvent-exposed with no contact with the pMHC, was intentionally replaced with glutamate. The resulting variant containing the additional F103E substitution was evaluated using Chai-1 as a semi-independent validation of the design.

### Cell lines

Human B-lymphoblastoid cell line 721.221 cells (ATCC CRL-1855), T2 cells (CRL-1992), and Jurkat T cells (ATCC TIB-152) were cultured in RPMI 1640 medium (Gibco, 72400047) supplemented with 10% fetal bovine serum (Gibco, 12483020). Cells were maintained at 37 °C in a 5% CO_2_ humidified incubator and passaged every 3 to 4 d to maintain exponential growth.

### Cell-binding assay by flow cytometry

Pulsed cells were washed one time with ice-cold fluorescence-activated cell sorting (FACS) buffer (PBS + 1% bovine serum albumin) and resuspended at 3.75 × 10^5^ to 5.00 × 10^5^ cells per 100 μl. Cells were stained with a final concentration of 50 nM preincubated HSA^DIII^-αRMF_V_H_ and fluorescein isothiocyanate (FITC) Anti-6X His tag antibody (Abcam, ab1206) for an hour at 4 °C in the dark. After the incubation, the stained cells were washed 2 times with the cold FACS buffer. After washing, cells were resuspended in 200 μl of the FACS buffer and analyzed on a CytoFLEX SRT cell sorter (Beckman Coulter). Data were analyzed using CytExpert SRT (Beckman Coulter). Intact cells were first gated on forward scatter area (FSC-A) versus side scatter area (SSC-A) to exclude debris (P1 gate), followed by single-cell selection using FSC-A versus forward scatter height (FSC-H) to eliminate doublets (P2 gate). Binding was quantified as mean fluorescence intensity within the live cell gate.

### Peptide pulsing

T2 and 721.221(HLA-A*02:01^+^/TAP^−^) cells were washed twice with serum-free RPMI 1640, resuspended at 1 × 10^6^ to 2 × 10^6^ cells/ml, and pulsed with peptides (RMF or RMF variants, 1 pM to 10 μM) in the presence of 10 μg/ml human β2M. The cells were transferred to low-attachment plates (SPL Life Sciences, 32006) and incubated for 18 to 22 h at 26 °C in a humidified incubator with 5% CO_2_ prior to subsequent functional assays. Pulsing with the p53^(R175H)^ peptide followed similar procedures.

### CAR-T activation assay

In each well of a 96-well v-bottom plate (SPL, 36296), 2.5 × 10^4^ HSA^DIII^-αRMF_V_H_ CAR-T cells were co-cultured with 2.5 × 10^4^ peptide-pulsed T2 or 721.221(HLA-A*02:01^+^/TAP^−^) in RPMI 1640 supplemented with 10% fetal bovine serum. The co-cultures were incubated for 24 h at 37 °C in a humidified incubator with 5% CO_2_. Following incubation, the culture supernatant was collected to measure interleukin-2 (IL-2) secretion using a human IL-2 enzyme-linked immunosorbent assay (ELISA) kit (BioLegend, 431815) according to the manufacturer’s instructions. The 3,3′,5,5′-tetramethyl benzidine substrate was incubated for 20 min, and absorbance was recorded at 450 nm using a Hidex Sense microplate reader (Hidex).

### Generation of stable cell lines

721.221(HLA-A*02:01^+^/TAP^−^) was generated through sequential transduction using lentiviral knock-in and lentivirus-mediated CRISPR/Cas9 knock-out system. In brief, a gene block encoding HLA-A*02:01^+^ (IDT) was cloned into pLV-EF1a-IRES-Puro lentiviral vector (Addgene #85132). The pLV-EF1a-HLA-A*02:01^+^-IRES-Puromycin construct was cotransfected with lentiviral packaging plasmids pMD2.2 (Addgene #12259), pRSV-REV (Addgene #12253), and pMDL g/p PRE (Addgene #12251) into HEK293T cells (ATCC CRL-3216). At 48 h posttransfection, the viral supernatant was exchanged with RPMI 1640 and concentrated to a final volume of approximately 1 ml using Amicon Ultra centrifugal filters (Millipore, UFC9050). The concentrated viral medium was filtered through a 0.45-μm-pore polyethersulfone filter (Sartorius, 16533) before use. For transduction, 721.221 cells were plated into a 6-well plate 1 d prior to viral transduction. One milliliter of the prepared viral particles was added dropwise to the cells in the presence of 8 μg/ml sequabrene. At 24 h posttransduction, cells were selected with 5 μg/ml puromycin. The selection medium was refreshed every 2 to 3 d until the cell population reached approximately 1 × 10^5^ cells. For single-cell clone generation, cells were serially diluted and plated into 96-well plates and then incubated until colony formation. The expression of HLA-A*02:01 was confirmed by flow cytometry using phycoerythrin-conjugated anti-human HLA-A,B,C antibody (BioLegend, #311406). Following the selection of HLA-A*02:01^+^ cells, TAP1 was knocked out using a lentiviral CRISPR/Cas9 system. A guide RNA targeting TAP1 (5′-GTTGGCCCGAGCATTGATCCGG-3′), located on chromosome 6, was cloned into a lentiCRISPRv2-blast vector (Addgene #98293). HEK293T cells were transfected with this construct to produce viral particles, which were subsequently used to infect HLA-A*02:01^+^ 721.221 cells. Transduced cells were selected with 5 μg/ml blasticidin and subjected to single-cell cloning via serial dilution to establish stable knock-out lines.

### Generation of the HSA^DIII^-αRMF_V_H_ CAR-T cell line

A second-generation CAR construct was generated by fusing the HSA^DIII^ with GS linker to the CD8α hinge and transmembrane domains, in sequence with the intracellular signaling domains of 4-1BB and CD3ζ. A truncated low-affinity nerve growth factorreceptor (ΔLNGFR; CD271) was included as a selection marker. The CAR cassette was cloned into a pLV-EF1a-IRES-Puro lentiviral vector by replacing the IRES-Puro element with p2A-ΔLNGFR. The lentiviral particles produced by the CAR transfer vectors transduced Jurkat cells. After 72 h of transduction, ΔLNGFR-positive cells were enriched using anti-CD271 MicroBeads and MACS columns (Miltenyi Biotec) according to the manufacturer’s instructions. The CAR expression was confirmed by flow cytometry using CD271(LNGFR)-FITC Antibody (Miltenyi Biotec 130-112-605).

### Luciferase-based cytotoxicity assay

Luciferase-expressing THP-1(HLA-A*02^+^/WT1^+^) and HL-60(HLA-A*02^−^/WT1^+^) cells were used as target cells. Target cells were co-cultured with effector T cells at an effector-to-target ratio of 1:1 in the presence of TCE at concentrations ranging from 0.32 to 1,000 pM. After incubation for 24 h at 37 °C, target-cell viability was quantified using the Steady-Glo Luciferase Assay System (Promega) according to the manufacturer’s instructions. Luminescence was measured using a Hidex Sense microplate reader (Hidex). Specific lysis was calculated relative to target-plus-effector wells without TCE, which were defined as the 0% lysis control. Medium-only and effector-only wells were included to determine background luminescence. Concentration–response curves were fitted using a 4-parameter logistic model to estimate EC_50_ values.

## Results

### Structural information of an antibody bound to RMF peptide-loaded MHC

A TCR-like antibody against the RMF/HLA-A*02 complex, named Q2L, was reported without structural information [24]. We determined the crystal structure of the Q2L-Fab bound to RMF/HLA-A*02 at a 3.0-Å resolution (Table 1).

**Table 1.** Data collection and refinement statistics

Data collection	
Space group	*P*2_1_2_1_2_1_
Unit cell dimensions	
*a*, *b*, *c* (Å)	101.07, 132.81, 185.66
*α*, *β*, *γ* (°)	90.0, 90.0, 90.0
Wavelength (Å)	1.0000
Resolution (Å)	29.87–3.03 (3.18–3.03) ^a^
*R*-merge	0.1285 (1.505) ^a^
*I*/*σ*(*I*)	16.90 (1.74) ^a^
Completeness	99.83 (100) ^a^
Redundancy	13.5 (13.4) ^a^
Refinement	
No. of reflections	49,214 (4,834) ^a^
*R*_work_/*R*_free_ (%)	19.6/23.9
RMS deviations	
Bond (Å)/angle (°)	0.013/1.37
Average *B* values (Å^2^)	85.85
Ramachandran plot (%)	
Favored/additionally allowed	96.73/3.27
Outliers	0.0

RMS, root mean square

^a^ The numbers in parentheses are the statistics from the highest-resolution shell.

The asymmetric unit contains 2 molecules of Q2L-Fab/RMF/HLA-A*02.

Unexpectedly, the V_L_ domains are separated from their V_H_ counterparts, forming limited intermolecular interactions between the V_L_ domains of the 2 Fab molecules. These interactions are minor and likely represent crystal contacts rather than stable homodimeric interactions in solution. Furthermore, the V_L_ domain of one Fab molecule is positioned closer to the V_H_ domain of the other Fab than to its own V_H_. Consequently, each RMF/HLA-A*02 complex interacts with the V_H_ domain of one Fab molecule and the V_L_ domain of the other Fab molecule (Fig. 1). The latter interactions, involving His145 and Lys146 of HLA-A*02:01 and Ser50 and Leu53 of V_L_, are minor and unlikely to occur in solution.

**Fig. 1.**
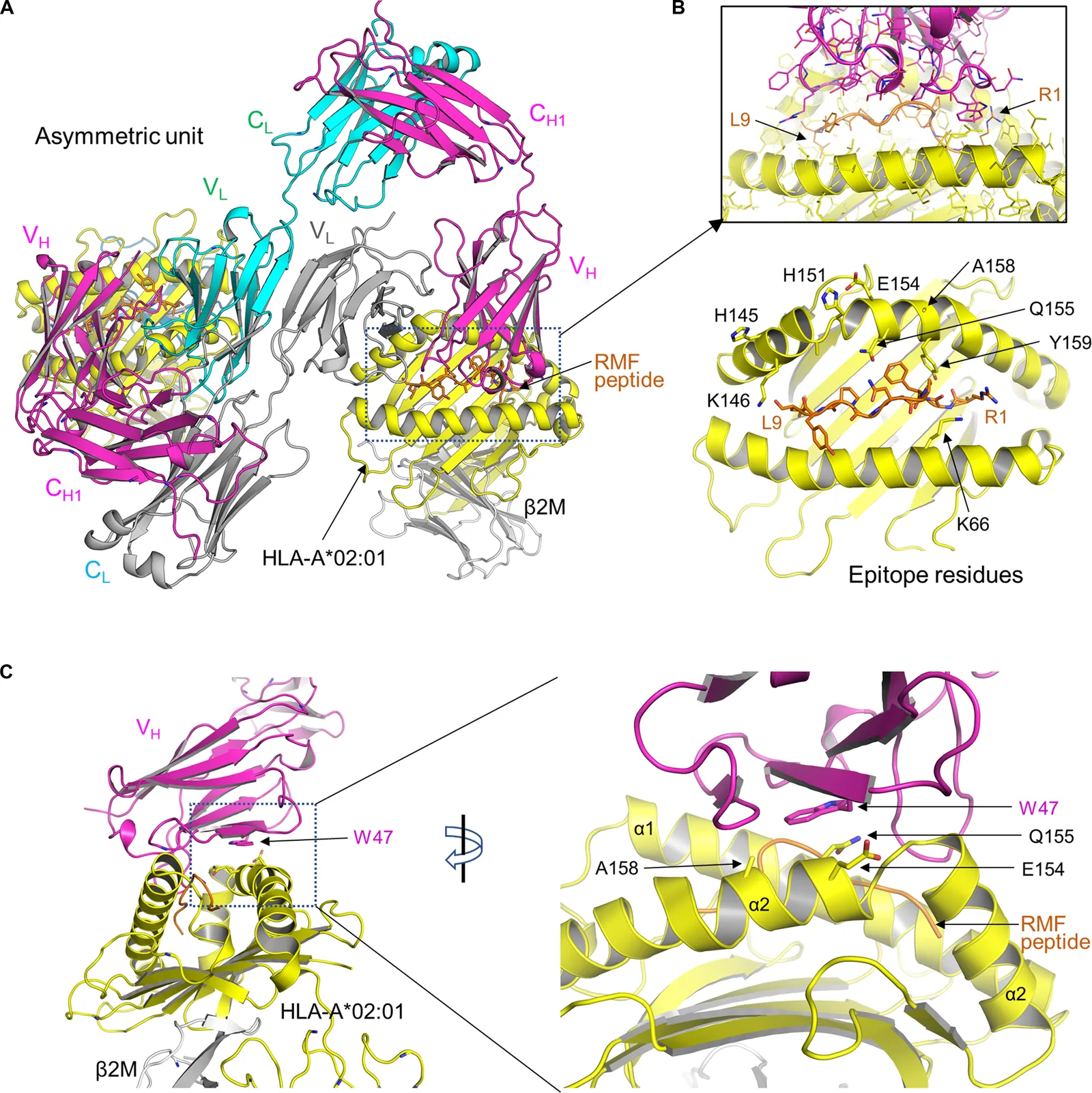
Structural features of Q2L-Fab/RMF/HLA-A*02. (A) Overall structure. Two molecules of the complex in the asymmetric unit are displayed. The V_L_ domain of one complex is separated from V_H_ and interacts with the V_L_ domain of the adjacent complex. The enlarged view shows that V_H_ spans the bound RMF peptide. (B) Peptide–major histocompatibility complex (pMHC) interaction with Fab. Peptide contacts with V_H_ are shown (top), and pMHC residues within 4 Å of the Fab (6 epitope residues) are shown as sticks and labeled (bottom). Also shown are His145 and Lys146 (in gray) from one complex, which interact with the V_L_ domain of the other Fab molecule. The detailed residue-level interactions are tabulated in Table S1. (C) Trp47 interaction with HLA-A*02:01. The indole ring of Trp47 in V_H_ sits on helix α2 (labeled) of HLA-A*02:01 and interacts with hydrocarbon atoms of Glu154, Gln155, and Ala158.

Typically, the framework regions of V_H_ and V_L_ create a hydrophobic interface between the 2 domains, and Q2L antibody preserves the conserved hydrophobic residues (Tyr36 and Phe98 of V_L_ and Leu45 and Phe103 of V_H_) that contribute to this interface formation. In the predicted Q2L structure, these residues are buried at the canonical V_H_–V_L_ interface. In the crystal structure, however, Tyr36 and Phe98 of V_L_ are only partially exposed and interact with each other due to an upward displacement of CDR3L toward the framework region. This conformation is further stabilized by the interaction with the other Fab molecule in the asymmetric unit (Fig. S1A). Leu45 and Phe103 of V_H_ are partially buried through contacts with the light chain of the other Fab molecule (Fig. S1A). We therefore posit that the V_H_ and V_L_ domains of this antibody associate with each other in isolation but dissociate from each other upon binding to RMF/HLA-A*02 through V_H_, enabling a unique binding mode that would otherwise be impossible due to severe steric clash between V_L_ and RMF/HLA-A*02. The observed unusual structural features demonstrate that the V_H_ domain of this antibody alone provides tight interactions with the pMHC. Two additional structural features stand out in the V_H_ interaction with RMF/HLA-A*02. First, V_H_ extensively contacts the bound RMF peptide while making limited contacts with only 6 HLA-A*02:01 residues (Fig. 1B, bottom). Second, the indole ring of Trp47 is positioned atop one of the “wall-forming” α-helices (α2) of the HLA-A*02:01, suggesting a potential anchoring role for pMHC docking (Fig. 1C). Trp47 is a highly conserved framework residue located on a β-strand in a common structural position within the V_H_ domain. We postulated that this docking configuration could be recapitulated by a V_H_ domain alone. If so, this sdAb and its docking position could serve as a common starting point for discovering TCR-like sdAbs that target other pMHCs primarily through peptide contacts, thereby reducing cross-reactivity with nontarget pMHCs.

### Design of a human V_H_ domain antibody binding to RMF/HLA-A*02

We then explored whether the V_H_ domain of Q2L could be produced in a soluble form and exhibit a similar binding affinity. However, the V_H_ domain (residues 1 to 115) was expressed in Chinese hamster ovary (CHO) cells as a soluble aggregate (Fig. S2). This was in sharp contrast with Q2L’s Fab that could be purified as a homogeneous monomeric form, indicating that the Q2L’s V_H_ alone is unstable. Previously, 5 point mutations introduced into trastuzumab yielded a soluble monomeric V_H_ domain (Tra V_H_), and its crystal structure was reported [33]. We sought to graft the key paratope residues of Q2L V_H_ onto Tra V_H_. A structural alignment of Tra V_H_ with Q2L V_H_/RMF/HLA-A*02 showed no backbone clashes with RMF/HLA-A*02, apart from its CDR3H loop (Fig. 2A), suggesting the possibility of converting it into a soluble V_H_ domain binding to RMF/HLA-A*02.

**Fig. 2.**
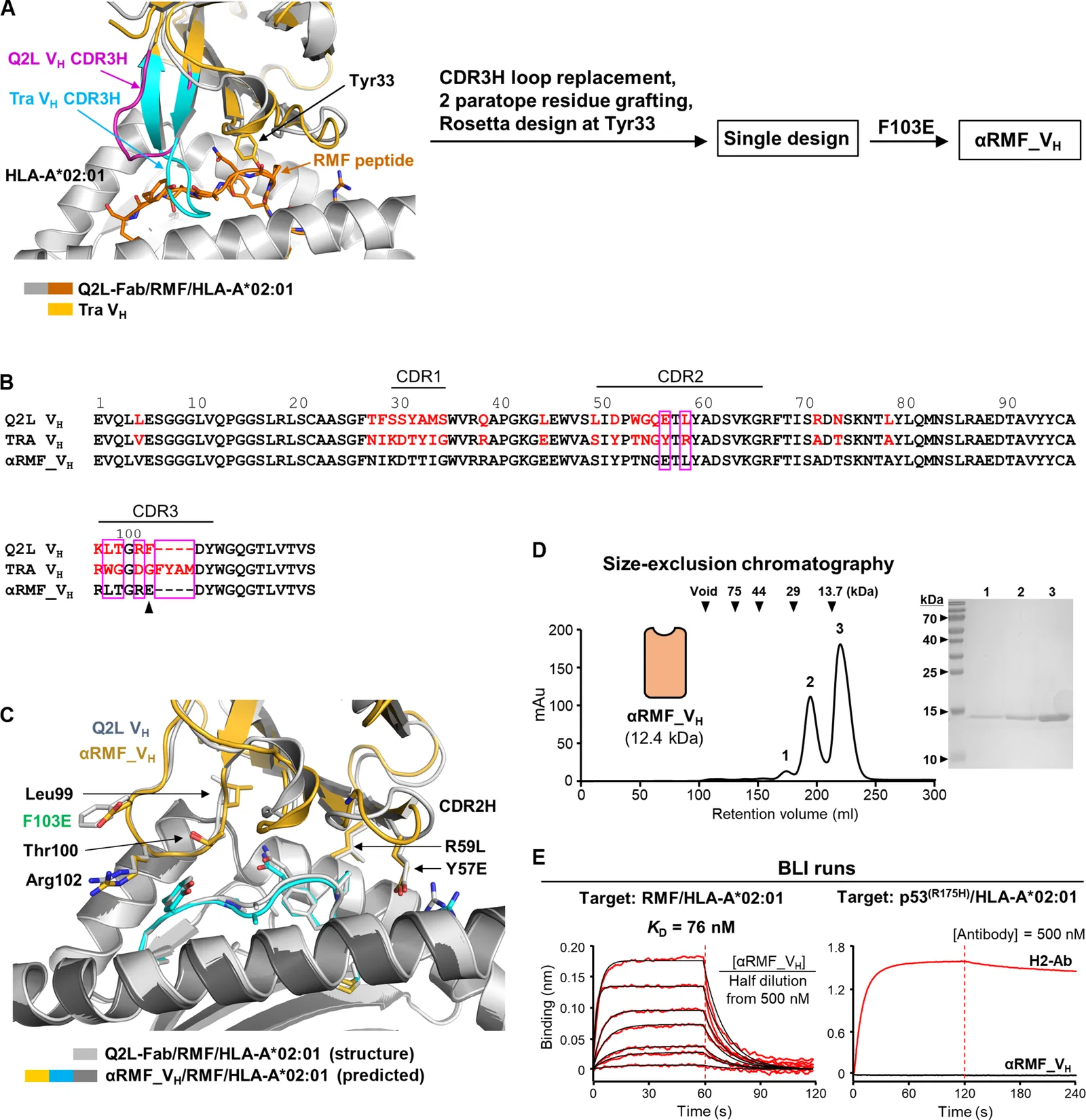
Design, purification, and binding affinity measurement of αRMF_V_H_. (A) Design procedure. Superposition of Tra V_H_ (PDB entry: 3B9V) onto Q2L-Fab/RMF/HLA-A*02 shows overall structural similarity. The CDR3H loops are highlighted in magenta or cyan and labeled. The longer CDR3H loop of Tra V_H_ clashes with RMF/HLA-A*02. Tyr33, which makes a steric clash with the bound peptide, is shown in sticks and labeled. The subsequent design steps are outlined. (B) Point mutations and deletions. The sequences of Q2L V_H_ and Tra V_H_ are aligned. Red letters indicate the residues that are different from each other. Pink boxes highlight the 5 point mutations in αRMF_V_H_ introduced based on the Q2L V_H_ sequence. The arrowhead marks the rationally replaced F103E mutation to avoid exposed hydrophobicity. (C) Superposition of a predicted structure of αRMF_V_H_/RMF/HLA-A*02 onto the Q2L-Fab/RMF/HLA-A*02 structure. The 5 key paratope residues occupy similar spatial positions (shown in sticks and labeled). Rational F103E mutation is shown and labeled in green letters. The RMF peptide in the predicted structure is shown in cyan. (D) Protein purification of αRMF_V_H_. The size-exclusion chromatography profile of αRMF_V_H_ on a Superdex 75 column is shown with molecular size markers. The inset shows sodium dodecyl sulfate–polyacrylamide gel electrophoresis (SDS-PAGE) analysis of the peak fractions. (E) Binding affinity and specificity of αRMF_V_H_. Biolayer interferometry (BLI) was performed by immobilizing 20 nM RMF/HLA-A*02 on streptavidin sensor tips, followed by association with monomeric αRMF_V_H_ and a dissociation phase. In parallel BLI experiments using immobilized p53^(R175H)^/HLA-A*02, H2-Ab showed binding, whereas αRMF_V_H_ showed no detectable binding.

After transplanting the CDR3H loop, grafting 2 paratope residues on CDR2H (Y57E and R59L), and introducing a Y33A mutation to relieve steric clashes, the model underwent Rosetta FastRelax [34], with only residue 33 designated for redesign. No substantial backbone shifts were observed, and threonine was most frequently enriched at this position. The final selected design included an additional rational F103E substitution to replace an exposed phenylalanine. This design, referred to as αRMF_V_H_, contains a total of 7 point mutations and a 4-residue deletion relative to the original Tra V_H_ sequence (Fig. 2B). Noting that Chai-1 [35] accurately predicts the Q2L V_H_/RMF/HLA-A*02 structure, we anticipated reliable modeling of αRMF_V_H_/RMF/HLA-A*02. The predicted αRMF_V_H_/RMF/HLA-A*02 model closely matched the Q2L V_H_/RMF/HLA-A*02 crystal structure, with key paratope residues occupying similar spatial positions (Fig. 2C and Fig. S3). Despite conformational differences in the CDR2H loop between Q2L V_H_ and Tra V_H_, the Y57E and R59L substitutions on this loop in αRMF_V_H_ remained compatible with RMF/HLA-A*02 (Fig. 2C).

For experimental validation, αRMF_V_H_ was expressed in *E. coli*. Size-exclusion chromatography showed that approximately 70% of the protein eluted as a monomer, with the remainder as a dimeric species (Fig. 2D). BLI affinity measurement showed the monomeric form bound RMF/HLA-A*02 with a dissociation constant (*K*_D_) of 76 nM (Fig. 2E). Next, to assess the specificity of αRMF_V_H_, we prepared HLA-A*02:01 loaded with p53^(R175H)^ peptide (HMTEVVRHC) and an Fv fragment, H2, known to bind this pMHC specifically [7]. Whereas the full antibody form of H2 (H2-Ab) bound strongly to p53^(R175H)^/HLA-A*02 in the BLI binding assay, αRMF_V_H_ showed no detectable interaction with this pMHC sharing the same HLA subtype (Fig. 2E).

### Construction and characterization of HSA fragment-fused αRMF_V_H_

To address the heterogeneous nature of αRMF_V_H_ (Fig. 2D), we fused HSA^DIII^, which is known to be highly soluble and to improve the solution behavior of fused client proteins [36,37]. The resulting fusion protein, HSA^DIII^-αRMF_V_H_, eluted from a size-exclusion chromatographic column as a single peak, corresponding to a monomeric protein size. BLI analysis showed that the fusion retained antigen-binding activity, with comparable *K*_D_ values for αRMF_V_H_ and HSA^DIII^-αRMF_V_H_ (76.4 and 81.4 nM, respectively) (Fig. 3A). These results indicate that HSA^DIII^ greatly improves the solution behavior of αRMF_V_H_ without affecting its binding affinity for RMF/HLA-A*02.

**Fig. 3.**
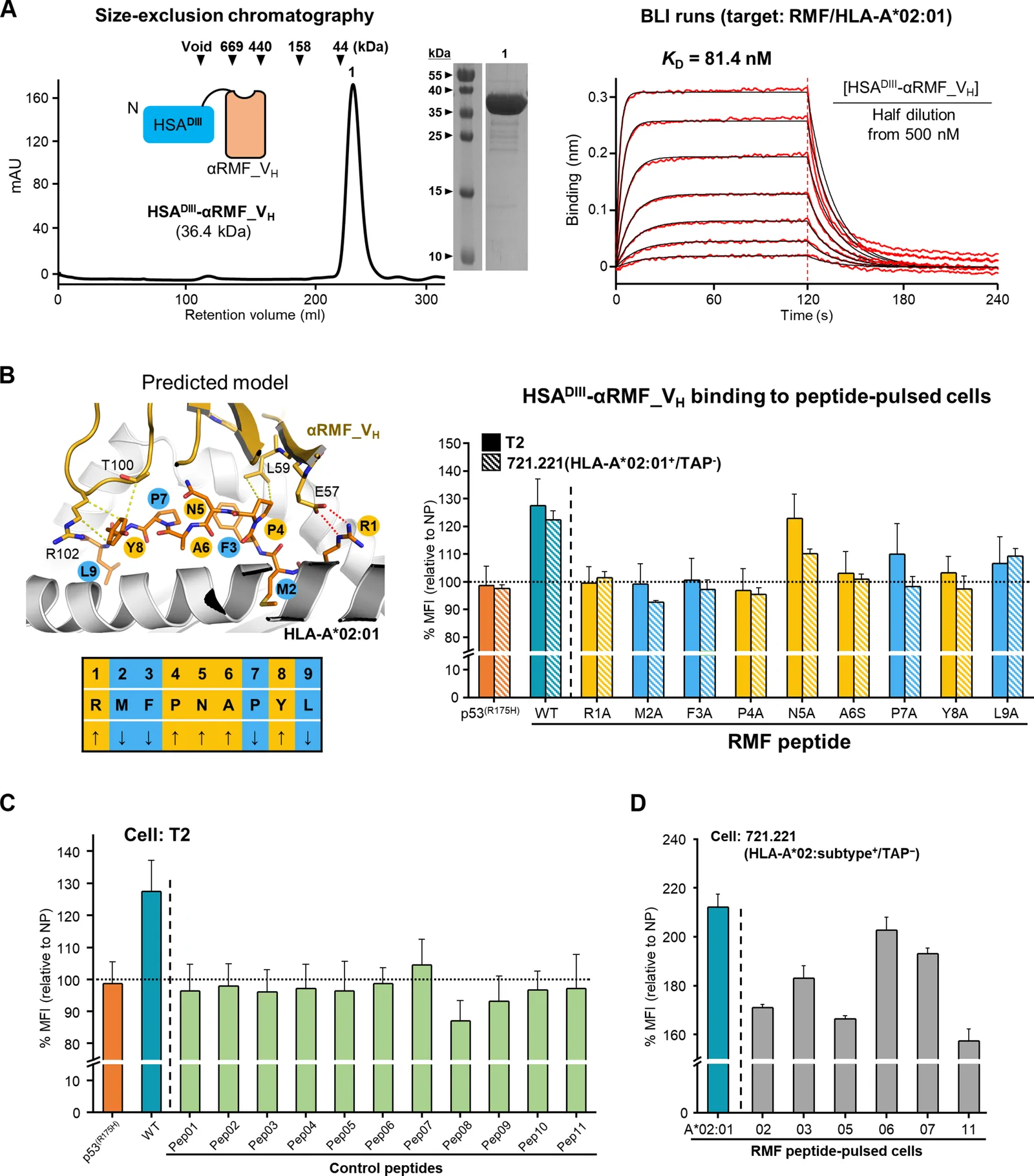
Properties of HSA^DIII^-αRMF_V_H_. (A) Purification and binding affinity. A schematic of the HSA^DIII^-αRMF_V_H_ fusion construct is shown. “N” stands for the N-terminus (top). The elution profile from Superdex 200 indicates the monodisperse nature of the fusion protein (left). Binding kinetics were measured by biolayer interferometry (BLI), using streptavidin (SA) sensor tips loaded with RMF/HLA-A*02, followed by incubation with HSA^DIII^-αRMF_V_H_ and the dissociation phase (right). (B) Alanine scanning of the RMF peptide and analysis of binding to cell-surface peptide–major histocompatibility complexes (pMHCs). Yellow and red dotted lines indicate key hydrophobic contacts (<4.1 Å) and ionic bonds, respectively, between the RMF peptide and αRMF_V_H_ in the structural model of αRMF_V_H_/RMF/HLA-A*02. The RMF peptide residues facing the V_H_ (↑) or HLA (↓) are color coded in yellow or blue, respectively (left). T2 and 721.221(HLA-A*02:01^+^/TAP^−^) cells were pulsed with 10 μM of the RMF peptide or the p53^(R175H)^ peptide, and then stained with fluorescein isothiocyanate (FITC)-labeled HSA^DIII^-αRMF_V_H_ (right). The *y*-axis represents the mean fluorescence intensity (MFI), expressed as a percentage relative to the unpulsed control (NP). The data represent the mean ± SD of 3 biological replicates. (C) Binding of HSA^DIII^-αRMF_V_H_ to cells pulsed with 11 control peptides. T2 cells were pulsed with p53^(R175H)^ peptide, RMF peptide, or the indicated 11 control peptides (Pep01 to Pep11) and then stained with FITC-labeled HSA^DIII^-αRMF_V_H_. The *y*-axis represents the MFI, expressed as a percentage relative to the unpulsed control (NP). Detailed sequences, source proteins, and provenance of the control peptides are provided in Table S4. The data represent the mean ± SD of 3 biological replicates. (D) Recognition of RMF presented by HLA-A*02 subtypes. Transporter associated with antigen processing (TAP)-deficient 721.221 cells expressing the indicated HLA-A*02 subtypes (A*02:01, A*02:02, A*02:03, A*02:05, A*02:06, A*02:07, and A*02:11) were pulsed with 10 μM RMF, and HSA^DIII^-αRMF_V_H_ binding was measured by flow cytometry. The *y*-axis represents the MFI, expressed as a percentage relative to the unpulsed control (NP). The data represent the mean ± SD of 3 biological replicates.

We next performed cell-binding assays using T2 and 721.221 cells to assess the specificity of HSA^DIII^-αRMF_V_H_ for peptide-loaded HLA-A*02:01 on the cell surface in 2 distinct cellular backgrounds. T2 is a human B/T-lymphoblastoid somatic hybrid that endogenously expresses HLA-A*02:01 and is naturally deficient in transporter associated with antigen processing (TAP). This deficiency allows cell-surface HLA-A*02:01 presentation to be efficiently controlled by exogenous peptide pulsing [38]. In contrast, 721.221 is a human B-lymphoblastoid line that lacks endogenous expression of HLA-A, HLA-B, and HLA-C but retains expression of TAP [39]. The modified 721.221(HLA-A*02:01^+^/TAP^−^) cell line was engineered to stably express HLA-A*02:01 in a TAP-deficient background. Although the 2 cell systems therefore provide a similar functional platform for exogenous peptide loading, they differ substantially in cellular background and in the origin and expression context of HLA-A*02:01. We used both systems to determine whether the binding specificity of HSA^DIII^-αRMF_V_H_ was reproducible across distinct cellular contexts rather than being attributable to a cell-line-specific effect.

Cells were pulsed with the RMF peptide, and cells interacting with HSA^DIII^-αRMF_V_H_ were analyzed by flow cytometry. In sharp contrast to unpulsed controls, a clear HSA^DIII^-αRMF_V_H_-positive population was observed in both RMF-pulsed T2 and 721.221(HLA-A*02:01^+^/TAP^−^) cells (Fig. 3B; right). In addition, consistent with the BLI binding analysis (Fig. 2E), HSA^DIII^-αRMF_V_H_ showed no detectable binding to either T2 or 721.221(HLA-A*02:01^+^/TAP^−^) cells pulsed with the p53^(R175H)^ peptide (Fig. 3B, right). To further assess HLA-A*02:01 restriction and exclude nonspecific binding to generic cell-surface components, we tested HSA^DIII^-αRMF_V_H_ binding to HLA-A*02:01-negative HL-60 cells with or without RMF peptide pulsing. No detectable binding was observed under either condition (Fig. S4), consistent with HLA-A*02:01-dependent recognition and suggesting minimal nonspecific binding to cell-surface components under these assay conditions.

### Mutational mapping of the RMF epitope and HSA^DIII^-αRMF_V_H_ paratope

To identify RMF peptide residues critical for binding and to assess the potential cross-reactivity of HSA^DIII^-αRMF_V_H_ to a similar peptide on the same HLA, cell-binding experiments were performed using RMF peptides containing positional alanine substitutions (RMF_1A to RMF_9A, except RMF_6S with an Ala-to-Ser substitution). The p53^(R175H)^ peptide was used as a negative control. Flow cytometry analysis revealed that substitutions at Arg1, Met2, Pro4, or Tyr8 significantly diminished HSA^DIII^-αRMF_V_H_ binding, indicating that these residues are important for recognition (*P* values in Table S2). Consistently, in the predicted structure of αRMF_V_H_/RMF/HLA-A*02, these peptide residues, except Met2, engage in hydrophobic or ionic interactions with αRMF_V_H_ (Fig. 3B). In the Q2L-Fab/RMF/HLA-A*02 structure, Met2 is deeply buried within the HLA groove, interacting with Tyr7, Phe9, Met45, Val67, His70, and Tyr99 of HLA-A*02:01. Although Met2 is inaccessible to the antibody, the M2A substitution likely disrupts the peptide binding to HLA-A*02:01, thereby indirectly abolishing HSA^DIII^-αRMF_V_H_ interaction. Notably, none of the single substitutions enhanced binding over the wild-type RMF peptide. To distinguish the loss of direct paratope contacts from impaired peptide presentation, we assessed cell-surface HLA stabilization using the anti-HLA-A*02:01-specific antibody BB7.2 (Fig. S5). Except for the P2 anchor mutant M2A, all RMF variants stabilized surface HLA-A*02:01 to levels comparable to those of wild-type RMF, consistent with NetMHCpan 4.2 predictions (Table S3). These results support the interpretation that the reduced binding of the solvent-exposed variants primarily reflects the loss of specific paratope–epitope contacts, whereas the effect of M2A is mainly attributable to impaired peptide–HLA assembly.

To experimentally validate the αRMF_V_H_ design, we individually substituted 5 residues contacting RMF/HLA-A*02 with alanine and analyzed their binding to the pMHC complex by BLI (Fig. S6). E57A and L59A in CDR2H drastically reduced binding, while G101A or R102A in CDR3H substantially diminished binding signals. In contrast, T100A retained binding activity, consistent with the Thr100 side chain being oriented toward CDR3H rather than directly toward the pMHC. Together, these mutational data provide functional support for the αRMF_V_H_ design.

### Peptide and HLA subtype selectivity of HSA^DIII^-αRMF_V_H_

To further evaluate potential cross-reactivity with peptides presented by HLA-A*02:01, we assembled a panel of 11 peptides (Pep01 to Pep11) previously tested in studies of WT1/HLA-A*02:01 TCR-mimic antibodies [23,24] (Table S4). The panel included unrelated human or viral peptides (Pep01–Pep02, Pep05–Pep06, Pep08, and Pep11), sequence-similar peptides such as MED13L (Pep07) and HDAC1/2 (Pep09), and alternative WT1-derived epitopes or variants (Pep03–Pep04 and Pep10). T2 cells were pulsed with each peptide and analyzed for HSA^DIII^-αRMF_V_H_ binding by flow cytometry. Whereas the native RMF peptide produced a robust binding signal (~128% of NP), the p53^(R175H)^ control and all 11 test peptides remained near the unpulsed baseline (Fig. 3C). Apart from a slight signal with Pep07, none showed appreciable binding comparable to that induced by RMF, supporting preferential recognition of the target epitope over the unrelated and sequence-similar HLA-A*02:01-presented peptides tested.

To examine whether HSA^DIII^-αRMF_V_H_ recognizes the RMF peptide presented by other HLA-A*02 subtypes, TAP-deficient 721.221 cells expressing 1 of 7 HLA-A*02 subtypes (A*02:01, A*02:02, A*02:03, A*02:05, A*02:06, A*02:07, and A*02:11) were pulsed with the RMF peptide (Fig. 3D). HSA^DIII^-αRMF_V_H_ showed binding above the nonpulsed baseline for all subtypes tested, indicating peptide-dependent recognition across these A*02 subtypes. However, binding to the other subtypes was generally weaker than that to HLA-A*02:01.

### Activation of HSA^DIII^-αRMF_V_H_ CAR-T cells

To assess whether HSA^DIII^-αRMF_V_H_ can activate effector T cells in a CAR format, we engineered Jurkat T cells to express a CAR incorporating HSA^DIII^-αRMF_V_H_ (Fig. 4A). These CAR-T cells, designated as HSA^DIII^-αRMF_V_H_ CAR-T cells, were co-cultured with T2 cells pulsed with either the RMF peptide or the p53^(R175H)^ peptide, and subsequently IL-2 production was measured by ELISA. The RMF peptide induced a concentration-dependent increase in IL-2 starting at 10 nM (Fig. 4B), whereas the p53^(R175H)^ peptide did not elicit IL-2 production even at 10 μM. To further evaluate the specificity of activation, we repeated the experiment with 721.221(HLA-A*02:01^+^/TAP^−^) cells pulsed with the RMF peptide. Similar to the T2 cells, these cells produced IL-2 in a concentration-dependent manner when pulsed with the RMF peptide but not with the p53^(R175H)^ peptide (Fig. 4B). Together, these results demonstrate that the HSA^DIII^-αRMF_V_H_ CAR-T cells are selectively activated through specific recognition of the RMF/HLA-A*02 complex on the target cells.

**Fig. 4.**
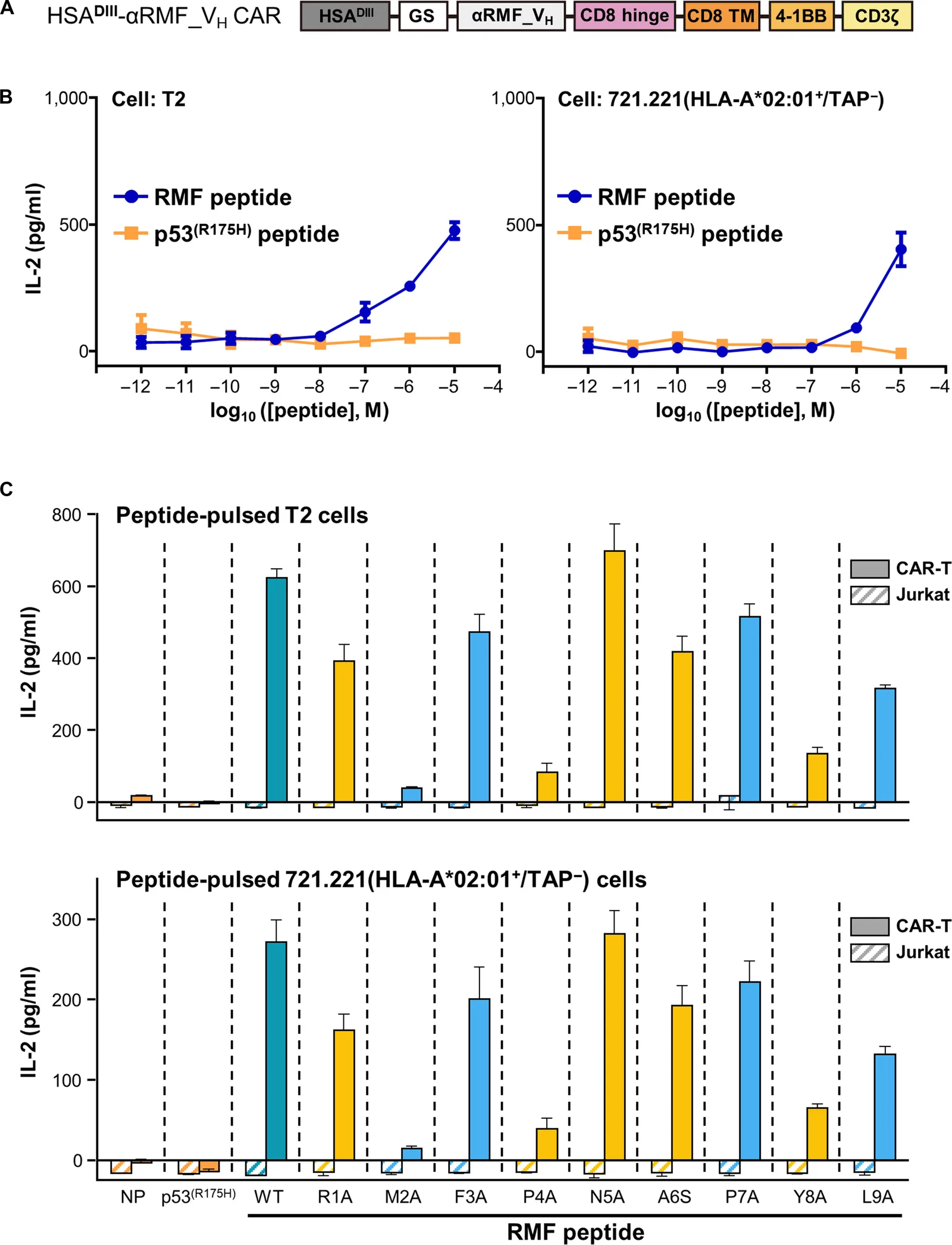
Activation of Jurkat T cells expressing HSA^DIII^-αRMF_V_H_ chimeric antigen receptor (CAR). (A) Schematic of the CAR construct. GS stands for GlySer sequence, and TM stands for transmembrane region. (B) Activation by the RMF peptide. T2 cells (left) and 721.221(HLA-A*02:01^+^/TAP^−^) cells (right) were pulsed with various concentrations of RMF or p53^(R175H)^ peptides and coincubated with HSA^DIII^-αRMF_V_H_ CAR-T cells at a 1:1 ratio. The graphs show the measurement of interleukin-2 (IL-2) release as a function of peptide concentration. The data represent the mean ± SD of 3 biological replicates. (C) Diminished activation by mutated RMF peptides. The top panel shows T2 cells, and the bottom panel shows 721.221(HLA-A*02:01^+^/TAP^−^) cells, each pulsed with 10 μM of the indicated RMF peptides. These cells were coincubated with HSA^DIII^-αRMF_V_H_ CAR-T cells at an effector cell:target cell ratio of 1:1. Controls included unmodified Jurkat T cells and cells pulsed with the p53^(R175H)^ peptide, while NP indicates unpulsed control.

To evaluate whether the activation of HSA^DIII^-αRMF_V_H_ CAR-T cells correlates with the peptide-recognition pattern of HSA^DIII^-αRMF_V_H_ for the RMF peptide, we repeated the experiment using T2 cells and 721.221(HLA-A*02:01^+^/TAP^−^) cells pulsed with a panel of peptides, RMF_1A through RMF_9A and RMF_6S. Unmodified Jurkat T cells used as a control showed no activation by any peptide. All mutant peptides showed reduced activation relative to the wild-type RMF peptide in both cell lines with the exception of N5A (Fig. 4C; *P* values in Table S5). The extent of this reduced activation paralleled the qualitative binding level of HSA^DIII^-αRMF_V_H_ for RMF/HLA-A*02 (Fig. 3B), because alanine substitutions at key residue positions (M2A, P4A, and Y8A) resulted in significantly decreased activation. In unpulsed controls, the HSA^DIII^-αRMF_V_H_ CAR-T cells showed no activation on either T2 (which expresses all class I HLAs) or 721.221 cells, demonstrating that HSA^DIII^-αRMF_V_H_ does not cross-react with empty HLAs or other cell-surface components.

### Cytotoxicity of αRMF_V_H_-HSA^DIII^-αRMF_V_H_ in TCE format

To further enhance the binding strength of HSA^DIII^-αRMF_V_H_, we constructed a bivalent variant. In this construct, the αRMF_V_H_ molecule was attached to both the N- and C-termini of HSA^DIII^, yielding a bivalent fusion protein denoted αRMF_V_H_-HSA^DIII^-αRMF_V_H_. The bivalent fusion protein was expressed well and purified as a monodisperse monomer by size-exclusion chromatography (Fig. 5A). This result indicated that adding an additional αRMF_V_H_ molecule does not induce aggregation, enabling the construct to maintain a stable, monomeric state.

**Fig. 5.**
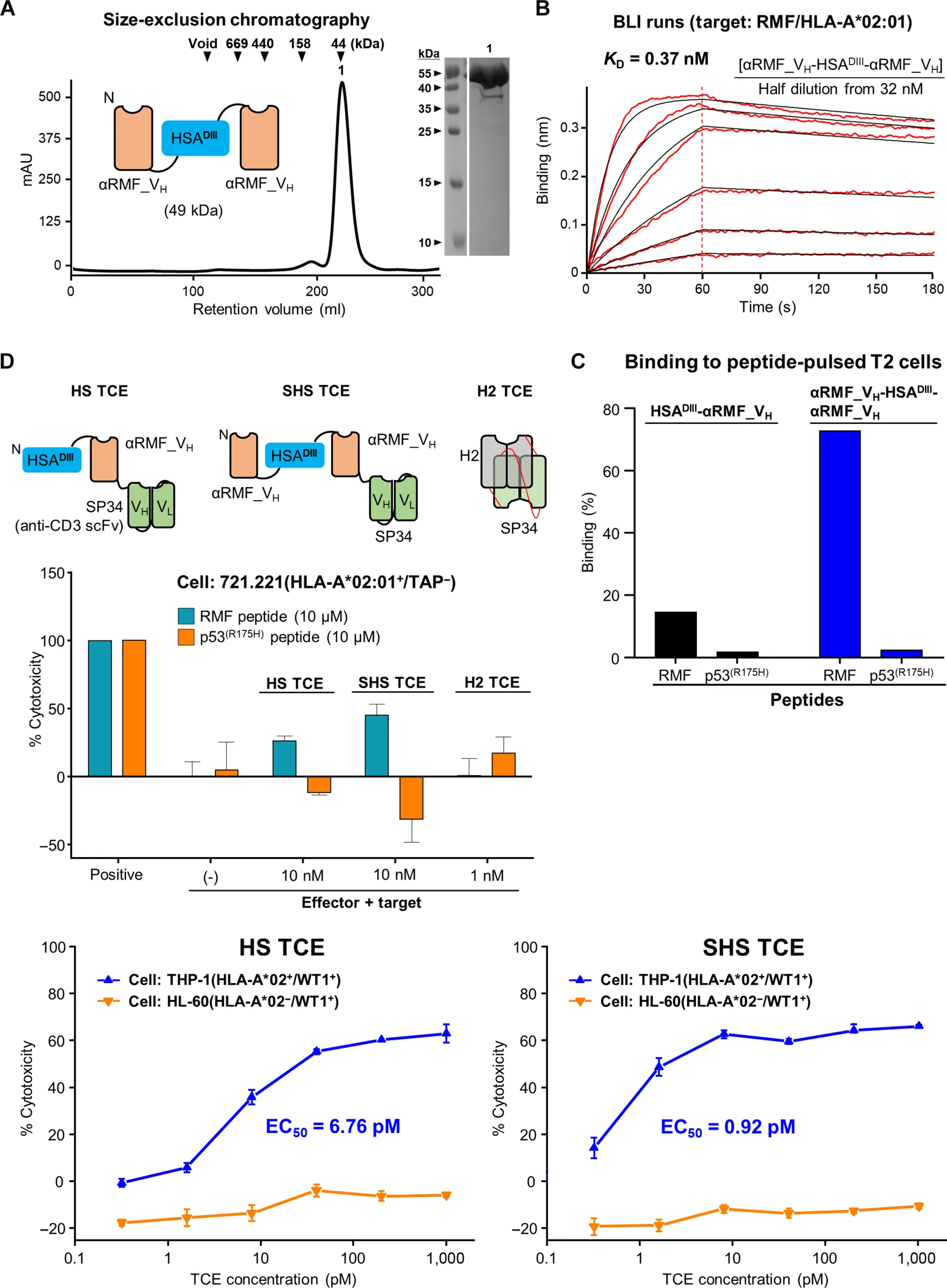
Enhanced antigen-binding affinity and cytotoxicity of αRMF_V_H_-HSA^DIII^-αRMF_V_H_. (A) Schematic and Superdex 200 elution profile of the fusion construct, which was produced and purified as a monomer. (B) Binding avidity measurement. Streptavidin (SA) sensor tips were loaded with biotinylated RMF/HLA-A*02, followed by incubation with the fusion construct and the dissociation phase. Sensorgrams were fitted using a 2:1 bivalent analyte binding model, yielding an apparent *K*_D_ of 0.37 nM. (C) Compared to the monovalent HSA^DIII^-αRMF_V_H_, the bivalent construct (αRMF_V_H_-HSA^DIII^-αRMF_V_H_) exhibited binding capacity to T2 cells pulsed with the RMF peptide (10 μM), but not to T2 cells pulsed with the p53^(R175H)^ peptide (10 μM). The binding (%) indicates the percentages of the cells shifted from the gate assigned for unpulsed control. (D) Cytotoxicity of αRMF_V_H_-based T cell engagers (TCEs). HSA^DIII^-αRMF_V_H_ and αRMF_V_H_-HSA^DIII^-αRMF_V_H_ were fused to the anti-CD3 scFv SP34 to generate monovalent HSA^DIII^-αRMF_VH TCE (HS TCE) and bivalent αRMF_VH-HSA^DIII^-αRMF_VH TCE (SHS TCE), respectively. As a comparator, H2 TCE was used, which is composed of anti-p53^(R175H)^/HLA-A*02 Fv and SP34 in a single-domain diabody format. (Top) Schematic representations of the 3 TCE constructs. (Middle) Peptide-dependent cytotoxicity against 721.221(HLA-A*02:01^+^/TAP^−^) cells pulsed with the indicated peptides. Target cells were co-cultured with T cells at an effector-to-target ratio of 1:1 for 4 h. Cell lysis was measured by calcein-AM release assay. HS and SHS TCEs selectively killed RMF-pulsed cells, with SHS TCE showing greater cytotoxicity, whereas H2 TCE selectively killed p53^(R175H)^-pulsed cells. (Bottom) Cytotoxicity against THP-1(HLA-A*02:01^+^/WT1^+^) and HL-60(HLA-A*02:01^−^/WT1^+^) cells was measured using a 24-h luciferase-based assay. Both HS and SHS TCEs induced concentration-dependent killing of THP-1 cells, with greater potency observed for SHS TCE, whereas neither induced detectable cytotoxicity against HL-60 cells. Data are shown as mean ± SD of triplicate wells.

BLI measurements showed that the bivalent αRMF_V_H_-HSA^DIII^-αRMF_V_H_ exhibited a dramatically enhanced apparent affinity for RMF/HLA-A*02, with an apparent *K*_D_ of 0.37 nM (Fig. 5B). This represents an ~200-fold increase in target binding relative to the monovalent HSA^DIII^-αRMF_V_H_, consistent with the expected avidity gain from dual antigen-binding domains. In agreement with the kinetic data, flow cytometry showed that the bivalent construct bound more strongly to T2 cells pulsed with the RMF peptide than did the monovalent construct (Fig. 5C). Importantly, this enhanced binding was antigen specific: the bivalent construct displayed no detectable binding to T2 cells pulsed with the p53^(R175H)^ peptide (Fig. 5C). Thus, the bivalent format greatly improved target-cell engagement through avidity while preserving the high specificity of the monovalent HSA^DIII^-αRMF_V_H_.

We next evaluated whether αRMF_V_H_ in TCE format could induce cytotoxic T-cell-mediated killing of target cells and whether the bivalency would translate to improved cytotoxicity. We constructed and produced monovalent TCE and bivalent TCE (Fig. 5D, top). Both TCEs showed target antigen-specific killing of peptide-pulsed 721.221(HLA-A*02:01^+^/TAP^−^) cells, with the bivalent TCE showing greater cytotoxicity in a 4-h calcein-AM release assay (Fig. 5D, middle). Next, their cytotoxicity was tested against 2 WT1-expressing leukemia cell lines: THP-1(HLA-A*02:01^+^/WT1^+^) expressing HLA-A*02:01 and HL-60(HLA-A*02:01^−^/WT1^+^) lacking the gene. THP-1(HLA-A*02:01^+^/WT1^+^) cells are expected to endogenously process WT1 and present the RMF peptide on the cell surface. The bivalent TCE induced concentration-dependent cytotoxicity against THP-1(HLA-A*02:01^+^/WT1^+^) cells but not against HL-60(HLA-A*02:01^−^/WT1^+^) cells (Fig. 5D, middle). The observed cytotoxicity was much weaker than that observed with the 721.221(HLA-A*02:01^+^/TAP^−^) cells, likely reflecting the short assay duration. We therefore assessed cytotoxicity over 24 h using a luciferase-based cytotoxicity assay. Both monovalent and bivalent TCEs induced robust, concentration-dependent killing of THP-1 cells, with EC_50_ values of 6.76 and 0.92 pM, respectively, while showing no notable cytotoxicity against HL-60 cells (Fig. 5D, bottom). These results demonstrate that αRMF_V_H_-based TCEs selectively kill cells presenting endogenously processed RMF/HLA-A*02:01 and that bivalency, designed to enhance target-binding avidity, substantially improves T-cell-mediated cytotoxicity.

## Discussion

For therapeutic applications, TCR-like antibodies must achieve exceptionally high specificity to minimize off-target pMHC engagement and cytotoxicity against healthy cells. This challenge arises from the conserved nature of MHC structures and the small surface area contributed by the displayed peptide, often making it difficult to isolate binders that are peptide specific yet tolerant of MHC diversity. This study addresses this challenge by designing a single-domain TCR-like antibody that preferentially recognizes the bound peptide while minimizing interactions with HLA.

The V_H_ domain of Q2L-Fab demonstrated peptide-centered binding to RMF/HLA-A*02, making it an attractive scaffold for conversion into a single-domain format. However, the isolated V_H_ was insoluble. To test whether a mutation cassette (H35G/Q39R/L45E/R50S) previously reported to improve V_H_ solubility [33] could confer solubility on Q2L V_H_, we introduced these mutations into Q2L V_H_. When expressed in *E. coli*, the resulting variant showed very low expression and was predominantly found in the insoluble fraction. We took an alternative discovery path of transplanting key paratope residues from Q2L V_H_ into the soluble Tra V_H_ scaffold. Through computational design, CDR3 transplantation and minimal mutations were introduced into a previously optimized monomeric V_H_ scaffold to discover an αRMF_V_H_ that binds RMF/HLA-A*02 with 2-digit nanomolar binding affinity. Structural comparison with the conventional TCR-mimic Fab ESK-1 (PDB ID: 4WUU) further highlights the distinct binding geometry of our engineered V_H_ domain (Fig. S7). Whereas ESK-1 approaches the pMHC surface at a steep, diagonal angle, contacting both the peptide and adjacent HLA α-helices, Q2L V_H_ adopts a markedly flatter orientation along the peptide-binding cleft.

αRMF_V_H_ fusing to HSA^DIII^ significantly improved solution behavior without compromising binding. Importantly, HSA^DIII^-αRMF_V_H_ exhibited high specificity in both BLI assays and cell-based assays, failing to bind to a closely related p53 mutant peptide presented on the same HLA subtype. Alanine scanning confirmed the functional relevance of several peptide residues, which aligned well with model-predicted paratope interactions, highlighting the peptide-centric nature of recognition. When expressed in CAR format, HSA^DIII^-αRMF_V_H_ triggered selective T cell activation in the presence of RMF peptide-loaded HLA-A*02:01, with no response to p53^(R175H)^ peptide-loaded or unpulsed cells. These findings suggest that αRMF_V_H_ may be applicable to engineered T cell approaches and provide additional evidence for the preferential recognition of the RMF/HLA-A*02:01 complex.

A nanobody-based TCE has demonstrated advantages over scFv-based TCEs, as the latter often suffer from suboptimal dual targeting due to potential mispairing between V_L_ and V_H_ domains and the necessity for a long connecting linker between the 2 scFv units [40]. Likewise, αRMF_V_H_ based on the soluble V_H_ scaffold derived from a human antibody could reduce the risk of off-target interactions compared to conventional Fabs, and its compact modularity allows straightforward fusion to other proteins or multivalent format construction as demonstrated with αRMF_V_H_-HSA^DIII^-αRMF_V_H_. Although isolated αRMF_V_H_ showed a dimeric fraction (~30%), this tendency was fully resolved upon HSA^DIII^ fusion. Further hydrophilic substitutions at exposed hydrophobic sites, such as those on the V_L_-facing surface in the Fab, may provide a rational strategy to improve the developability of αRMF_V_H_ as an isolated sdAb.

A previously reported TCR-like antibody against p53^(R175H)^/HLA-A*02, when converted into a TCE format, exhibited a *K*_D_ of 86 nM and effectively induced tumor cell killing and xenograft regression in mice [7]. HSA^DIII^-αRMF_V_H_ exhibits comparable affinity (*K*_D_ of 81 nM), and its bivalent form showed markedly enhanced target binding (*K*_D_ of 0.37 nM). Consistently, while both HSA^DIII^-αRMF_V_H_ and αRMF_V_H_-HSA^DIII^-αRMF_V_H_ in TCE format exhibited antigen-dependent cytotoxicity, the bivalent TCE exhibited much enhanced efficacy. Given that the RMF peptide is derived from the oncogenic WT1 protein, aberrantly expressed in leukemia and multiple solid tumors, αRMF_V_H_ may provide a foundational tool for diverse therapeutic formats, including bispecific antibodies, TCEs and antibody–drug conjugates.

Recent studies have reported complementary computational strategies for antibody engineering, including generative models for sequence and structure design and BioPhi-guided humanness optimization coupled with structure prediction, docking, and molecular dynamics [41,42]. Our approach differs from conventional library screening or *in vitro* evolution by leveraging high-resolution structural data and computational design to construct a TCR-like sdAb. Furthermore, the structural features and design strategy described here can serve as a generalizable blueprint for developing sdAbs against other clinically relevant pMHC targets. In conclusion, this work establishes a peptide-focused, computational design platform for generating highly specific, TCR-like sdAbs in a compact and stable format, expanding the repertoire of molecules available for targeting intracellular antigens presented on MHC I.

## Data Availability

The coordinates of the Q2L-Fab/RMF/HLA-A*02 structures have been deposited in the Protein Data Bank (PDB ID: 9VSO).
