# Supplementary material for: Computational Design of a TCR-like Human VH Domain Antibody That Specifically Recognizes a WT1-Derived Peptide Presented by Class I Major Histocompatibility Complex
Source: Comput Struct Biotechnol J. 2026 Oct 15;35(1):0236. doi: 10.34133/csbj.0236 (PMC13669508; doi:10.34133/csbj.0236)
Supplement: Supplementary file 1 [file csbj.0236.f1.zip › Supplementary Materials.docx]

**Computational design of a TCR-like human V_H_ domain antibody that specifically recognizes WT1-derived peptide presented by class I MHC**

Tae-Sung Oh^1†^, Seong-Woo Kim^2†^, SangPhil Ahn^1^, Seonghyuk Suh^1^, JooYeon Lee^1^, YeongNam Bae^2^, Hyejin Lee^1^, Yong-Sung Kim^4^, Bo-Seong Jeong^1*^, Byung-Ha Oh^1,3*^

1. Therazyne, Inc., Daejeon, Republic of Korea.

2. Department of Biological Sciences, Korea Advanced Institute of Science and Technology, Daejeon, Republic of Korea.

3. Graduate Program of Engineering Biology, KAIST Institute for the Biocentury, Korea Advanced Institute of Science and Technology, Daejeon, Republic of Korea

4. Department of Molecular Science and Technology, Advanced College of Bioconvergence Engineering, Ajou University, Suwon, Republic of Korea.

*Corresponding author E-mail: jbs@therazyne.com and [bhoh@kaist.ac.kr](mailto:bhoh@kaist.ac.kr)

†These authors contributed equally to this work.

**Supplementary Figures**

**Figure S1. Structural environment of conserved interface residues in the crystal, steric incompatibility of the predicted Q2L Fab with RMF/HLA-A*02 and electron-density validation of the crystal.**

(A) Environment. The numbers in parentheses denote two molecules in the asymmetric unit. Tyr36 and Phe98 of V_L_ and Leu45 and Phe103 of V_H_ are partially buried by the other molecule in the asymmetric unit. The table shows the side-chain solvent-accessible surface area (SASA) of each residue in the crystal structure the corresponding SASA in a fully exposed state.

(B) Superposition of the predicted Q2L Fab onto the heavy chain of Q2L in the crystal structure, indicating severe steric clashes between the predicted V_L_ domain and the pMHC complex.

(C) 2*F*_o_–*F*_c_ electron-density maps around the V_L_/C_L_ linkage of the Q2L-Fab structure (*Left*) and the Q2L-Fab/RMF/HLA-A*02 interface (*Right*). The maps are shown as blue mesh and contoured at 1.0 σ.

**Figure S2. Size-exclusion chromatography and SDS-PAGE analysis of isolated Q2L V_H_ expressed in CHO cells.**

Q2L V_H_ fraction collected from Ni^2+^ affinity chromatograph was loaded onto HiLoad 26/60 Superdex 75 column. The size marker positions are shown by triangles. The chromatogram shows that Q2L V_H_ was not expressed as a monodisperse species of the expected monomeric size. Fractions corresponding to the indicated peaks were analyzed by SDS-PAGE.

**Figure S3. Structural alignment and residue-level contact analysis of Q2L V_H_ in the crystal and designed αRMF_V_H_.**

The designed αRMF_V_H_/RMF/HLA-A*02 model was superimposed onto Q2L Fab/RMF/HLA-A*02. For clarity, the design model of pMHC is omitted. The RMSD for all superimposed V_H_ Cα atoms is 0.42 Å. The table lists the contacting residue pairs (heavy-atom distance cutoff ≤ 4.3 Å).

**Figure S4. Cell-surface binding to HLA-A*02-positive T2 cells and HLA-A*02-negative HL-60 cells.**

Cells were pulsed with RMF or p53^(R175H)^ peptide and analyzed for HSA^DIII^–αRMF_V_H_ binding by flow cytometry. MFI values were normalized to the corresponding non-pulsed control. RMF increased binding to T2 cells but not to HL-60 cells. Data represent the mean ± SD of three independent biological experiments.

**Figure S5. Assessment of HLA-A*02 stabilization by WT RMF and RMF variants.**

T2 and 721.221(HLA-A*02:01⁺/TAP⁻) cells were pulsed with 10 μM p53^(R175H)^, WT RMF, or the indicated RMF variants in the presence of human β2M. The presence of intact HLA-A*02 on the cell surface was assessed by flow cytometry using the BB7.2 antibody. MFI was normalized to the corresponding non-pulsed control (NP = 100%; dotted line). The data represent the mean ± SD of three biological replicates.

**Figure S6. BLI analysis of αRMF_V_H_ paratope mutants binding to RMF/HLA-A*02.**

Biotinylated RMF/HLA-A*02 complex was immobilized onto a streptavidin biosensor tips. Parental αRMF_V_H_ and the five indicated alanine-substituted variants were tested as analytes at 100 nM. E57A and L59A showed barely detectable binding, whereas G101A and R102A showed reduced responses, and T100A retained binding.

**Figure S7. Structural comparison of RMF/HLA-A*02 recognition by ESK-1 and Q2L V_H_.**

The crystal structures of the ESK-1 Fab–RMF/HLA-A*02 complex (PDB ID: 4WUU; *Left*) and the Q2L V_H_–RMF/HLA-A*02 complex (*Right*) are shown in the same orientation after alignment of HLA-A*02:01. ESK-1 exhibits a more diagonal docking mode over the pMHC surface, whereas Q2L V_H_ binds in a flatter orientation, resulting in distinct peptide-contact interactions.

**Supplementary Tables**

| **Contact Category** | **Q2L Paratope Residue** | **Epitope**  **Residues** | **Distance (Å)** | **Interaction Type** |
| --- | --- | --- | --- | --- |
| **Intra-complex contacts** | CDR1H / Ala33 | RMF / Pro4 | 4.16 | Van der Waals |
|  | CDR2H / Glu57 | RMF / Arg1 | 3.24 | Salt bridge |
|  | CDR2H / Leu59 | RMF / Pro4 | 3.69 | Hydrophobic contact |
|  | CDR3H / Thr100 | RMF / Pro7 | 3.25 | Van der Waals contact |
|  | CDR3H / Thr100 | RMF / Tyr8 | 3.04 | Hydrogen bond |
|  | Framework / Trp47 | HLA / Glu154 | 3.32 | Hydrophobic contact |
|  | Framework / Trp47 | HLA / Gln155 | 3.60 | Hydrophobic contact |
|  | Framework / Trp47 | HLA / Ala158 | 3.74 | Hydrophobic contact |
| **Inter-complex contacts** | V_L_ / Ser50 | HLA / His145 | 2.77 | Hydrogen bond |
|  | V_L_ / Leu53 | HLA / Lys146 | 3.86 | Van der Waals contact |

**Table S1. Residue-level contacts between Q2L Fab and RMF/HLA-A*02:01 in the crystal structure.**

Interatomic contacts were analyzed using the Q2L Fab/RMF/HLA-A02 crystal structure. Intra-complex contacts comprise interactions of the cognate Q2L V_H_ with the RMF peptide or HLA-A*02:01, whereas inter-complex contacts involve the V_L_ domain of an adjacent Fab molecule and are interpreted as crystal-packing contacts. Hydrogen bonds and salt bridges were identified based on polar or charged atom geometry. Hydrophobic contacts denote interactions between extended nonpolar side-chain regions, whereas van der Waals contacts denote localized nonbonded packing between a specific pair of heavy atoms.

| **Cell line** | **Mutation in RMF peptide** | **Mean difference (variant − WT)** | **Lower**  **95% CI** | **Upper**  **95% CI** | **Dunnett-adjusted**  ***p* value** | **Significance** |
| --- | --- | --- | --- | --- | --- | --- |
| T2 | R1A | -27.93 | -35.63 | -20.24 | 0.0000000089 | **** |
|  | M2A | -28.28 | -35.97 | -20.58 | 0.0000000067 | **** |
|  | F3A | -26.84 | -34.54 | -19.15 | 0.0000000213 | **** |
|  | P4A | -30.49 | -38.18 | -22.79 | 0.0000000011 | **** |
|  | N5A | -4.58 | -12.28 | 3.11 | 0.4144300000 | ns |
|  | A6S | -24.48 | -32.18 | -16.79 | 0.0000001300 | **** |
|  | P7A | -17.44 | -25.13 | -9.74 | 0.0000185260 | **** |
|  | Y8A | -24.15 | -31.85 | -16.46 | 0.0000001660 | **** |
|  | L9A | -20.80 | -28.50 | -13.11 | 0.0000017785 | **** |
| 721.221 | R1A | -18.29 | -24.79 | -11.80 | 0.0000009785 | **** |
|  | M2A | -25.89 | -32.39 | -19.40 | 0.0000000009 | **** |
|  | F3A | -22.02 | -28.52 | -15.53 | 0.0000000385 | **** |
|  | P4A | -23.50 | -29.99 | -17.00 | 0.0000000096 | **** |
|  | N5A | -10.71 | -17.20 | -4.21 | 0.0008374370 | *** |
|  | A6S | -18.75 | -25.24 | -12.25 | 0.0000006730 | **** |
|  | P7A | -21.04 | -27.54 | -14.54 | 0.0000000935 | **** |
|  | Y8A | -21.85 | -28.35 | -15.35 | 0.0000000450 | **** |
|  | L9A | -11.44 | -17.93 | -4.94 | 0.0004091390 | *** |

**Table S2. Statistical analysis of HSA^DIII^– αRMF_V_H_ binding to cell-surface pMHC complexes.**

Binding data obtained with T2 and 721.221(HLA-A*02:01⁺/TAP⁻) cells (Figure 3B) were analyzed by one-way repeated-measures ANOVA with Greenhouse–Geisser correction, followed by Dunnett’s multiple-comparisons test against WT RMF peptide. Significant overall effects of peptide substitution were observed in T2 cells [F(1.851,3.702)=32.211, *p*=0.00468] and 721.221 cells [F(1.622,3.244)=25.654, *p*=0.0108]. Mean differences are reported as variant minus WT, together with 95% confidence intervals and Dunnett-adjusted *p*-values. Negative values indicate reduced binding relative to WT RMF peptide. All variants except N5A showed significantly reduced binding in T2 cells, whereas all tested variants showed significant reductions in 721.221 cells. Analyses were based on three independent biological experiments. The p53^(R175H)^ negative control was not included in the positional-variant ANOVA. A Dunnett-adjusted *p*<0.05 was considered statistically significant (∗: *p*<0.05, ∗∗: *p*<0.01, ∗∗∗: *p*<0.001, ∗∗∗∗: *p*<0.0001, ns: not significant).

| **Variant** | **Sequence** | **%Rank_EL** | **EL class** | **%Rank_BA** | **Predicted affinity (nM)** | **BA class** |
| --- | --- | --- | --- | --- | --- | --- |
| WT RMF | RMFPNAPYL | 0.021 | SB | 0.050 | 5.58 | SB |
| R1A | AMFPNAPYL | 0.017 | SB | 0.070 | 6.84 | SB |
| M2A | RAFPNAPYL | 0.610 | WB | 3.021 | 576.81 | NB |
| F3A | RMAPNAPYL | 0.053 | SB | 0.151 | 11.20 | SB |
| P4A | RMFANAPYL | 0.095 | SB | 0.033 | 5.01 | SB |
| N5A | RMFPAAPYL | 0.027 | SB | 0.033 | 4.98 | SB |
| A6S | RMFPNSPYL | 0.015 | SB | 0.032 | 4.92 | SB |
| P7A | RMFPNAAYL | 0.082 | SB | 0.073 | 6.98 | SB |
| Y8A | RMFPNAPAL | 0.054 | SB | 0.200 | 14.74 | SB |
| L9A | RMFPNAPYA | 0.111 | SB | 0.167 | 12.35 | SB |
| p53^(R175H)^ | HMTEVVRHC | 1.761 | WB | 9.057 | 4,543.17 | NB |

**Table S3. NetMHCpan prediction of HLA-A*02:01 binding by RMF positional variants.**

WT RMF, the indicated single-position RMF variants, and the p53^(R175H)^ control peptide were analyzed using NetMHCpan 4.2. Eluted-ligand (EL) and binding-affinity (BA) scores, percentile ranks, predicted affinity values, and binding classifications are shown. Peptides with %Rank_EL ≤ 0.5 and ≤ 2.0 were classified as strong binders (SB) and weak binders (WB), respectively. All RMF variants except M2A were predicted to be strong HLA-A*02:01 binders.

| **Peptide ID** | **Sequence** | **Source protein / gene** | **UniProt**  **accession** | **Residue range** | **Source study** |
| --- | --- | --- | --- | --- | --- |
| Pep01 | RIITSTILV | HuD / ELAVL4 | P26378 | 162-170 | Zhao et al. [24] |
| Pep02 | LLEEMFLTV | CDR2 | Q01850 | 290–298 |  |
| Pep03 | SLGEQQYSV | WT1 | P19544 | 187–195 |  |
| Pep04 | CMTWNQMNL | WT1 | P19544 | 235–243 |  |
| Pep05 | LMLGEFLKL | Survivin / BIRC5 | O15392 | 96–104 |  |
| Pep06 | FLTPKKLQCV | PSA / KLK3 | P07288 | 165–174 | Ataie et al. [23] |
| Pep07 | RMFPTPPSL | MED13L | Q71F56 | 863–871 |  |
| Pep08 | GLCTLVAML | EBV BMLF1 | Q04360 | 259–267 |  |
| Pep09 | RMLPHAPGV | HDAC1 / HDAC2 | Q13547 | 371–379 |  |
| Pep10 | YMFPNAPYL | RMF P1 variant (R1Y) | P19544 | 126–134 |  |
| Pep11 | SLYNTVATL | HIV-1 Gag | P03348 | 77–85 |  |

**Table S4. Composition and sources of the peptide panel used for αRMF_V_H_ selectivity test.**

The table lists the peptide identifier, amino-acid sequence, source protein or gene, UniProtKB accession, corresponding source-protein residue range, and literature source for each of the 11 candidate control peptides. Pep01–Pep05 were selected from the peptide panel reported by Zhao et al. [24], whereas Pep06–Pep11 were selected from the panel reported by Ataie et al. [23]. UniProtKB residue numbers refer to the indicated canonical source-protein sequence. For synthetic peptide analogues, the accession and residue range indicate the corresponding native protein region rather than an exact endogenous sequence.

| **Cell line** | **Mutation in RMF peptide** | **Mean difference (variant − WT)** | **Lower**  **95% CI** | **Upper**  **95% CI** | **Dunnett-adjusted**  ***p* value** | **Significance** |
| --- | --- | --- | --- | --- | --- | --- |
| T2 | R1A | -0.57 | -0.80 | -0.34 | 0.0000046047 | **** |
|  | M2A | -0.88 | -1.11 | -0.65 | 0.0000000017 | **** |
|  | F3A | -0.20 | -0.43 | 0.02 | 0.0895900000 | ns |
|  | P4A | -0.38 | -0.61 | -0.16 | 0.0006021550 | *** |
|  | N5A | 0.15 | -0.08 | 0.37 | 0.3280000000 | ns |
|  | A6S | -0.96 | -1.18 | -0.73 | 0.0000000002 | **** |
|  | P7A | -0.28 | -0.51 | -0.05 | 0.0113200000 | * |
|  | Y8A | -1.03 | -1.25 | -0.80 | 0.0000000000 | **** |
|  | L9A | -0.43 | -0.66 | -0.21 | 0.0001582290 | *** |
| 721.221 | R1A | -0.46 | -0.64 | -0.27 | 0.0000061891 | **** |
|  | M2A | -0.63 | -0.82 | -0.45 | 0.0000000297 | **** |
|  | F3A | -0.17 | -0.36 | 0.01 | 0.0738000000 | ns |
|  | P4A | -0.27 | -0.45 | -0.08 | 0.0031900000 | ** |
|  | N5A | 0.04 | -0.15 | 0.22 | 0.9957600000 | ns |
|  | A6S | -0.69 | -0.88 | -0.51 | 0.0000000044 | **** |
|  | P7A | -0.24 | -0.42 | -0.05 | 0.0079400000 | ** |
|  | Y8A | -0.74 | -0.92 | -0.55 | 0.0000000009 | **** |
|  | L9A | -0.37 | -0.55 | -0.18 | 0.0001020860 | *** |

**Table S5. Statistical analysis of RMF variant-induced HSA^DIII^–αRMF_V_H_ CAR-Jurkat activation.**

IL-2 secretion data obtained with peptide-pulsed T2 and 721.221(HLA-A*02:01⁺/TAP⁻) cells in Figure 4C were analyzed separately using one-way repeated-measures ANOVA with Greenhouse–Geisser correction, followed by Dunnett’s multiple-comparisons test against WT RMF. Significant overall effects of peptide substitution were observed in T2 cells [F(1.695,3.389)=55.715, *p*=0.00269] and 721.221 cells [F(1.774,3.547)=39.287, *p*=0.00395]. Mean differences are reported as variant minus WT, together with 95% confidence intervals and Dunnett-adjusted p values; negative values indicate reduced IL-2 production relative to WT RMF. R1A, M2A, P4A, A6S, P7A, Y8A, and L9A significantly reduced CAR-Jurkat activation in both target-cell systems, whereas F3A and N5A were not significantly different from WT RMF. Analyses were based on three independent biological experiments. The p53^(R175H)^ and unmodified Jurkat negative controls were not included in the positional-variant ANOVA. Figure 4C measures IL-2 secretion as the readout of antigen-dependent CAR-Jurkat activation. A Dunnett-adjusted *p* < 0.05 was considered statistically significant (*: *p* < 0.05, **: *p* < 0.01, ***: *p* < 0.001, ****: *p* < 0.0001, ns: not significant).
